# The effect of the metabolic syndrome on the risk and outcome of coronary artery bypass graft surgery

**DOI:** 10.5830/CVJA-2012-055

**Published:** 2012-08

**Authors:** Marius J Swart, Wihan H De Jager, Johann T Kemp, Paul J Nel, Sarel L Van Staden, Gina Joubert

**Affiliations:** Mediclinic Bloemfontein, South Africa; School of Medicine, University of the Free State, Bloemfontein, South Africa; School of Medicine, University of the Free State, Bloemfontein, South Africa; School of Medicine, University of the Free State, Bloemfontein, South Africa; School of Medicine, University of the Free State, Bloemfontein, South Africa; Department of Biostatistics, Faculty of Health Sciences, University of the Free State, Bloemfontein, South Africa

**Keywords:** CABG, metabolic syndrome

## Abstract

**Background:**

The individual components of the metabolic syndrome are risk factors for coronary artery disease. The underlying pathophysiology of a low-grade inflammatory process postulates that the metabolic syndrome could compromise a procedure such as coronary artery bypass graft surgery (CABG) done on cardiopulmonary bypass (CPB).

**Methods:**

From a single institution, 370 patients with the metabolic syndrome (IDF and ATP III criteria) and 503 patients without the metabolic syndrome were identified. The influence of the metabolic syndrome on the pre-operative core risk factors for CABG mortality as well as its effect on the mortality and major morbidity post surgery were investigated.

**Results:**

Patients with the metabolic syndrome were operated on less urgently than those without the metabolic syndrome. The EuroSCORE was also lower in those with the metabolic syndrome. Patients with the metabolic syndrome required fewer units of homologous red blood cells, but stayed statistically longer in hospital.

**Conclusions:**

In this surgical population the metabolic syndrome had no detrimental clinical effect on either the pre-operative risk factors or the outcome after CABG.

## Abstract

Major surgery such as coronary artery bypass graft surgery (CABG) has the risk of mortality and morbidity. Co-morbidities will contribute to this potential risk of complications. In the well-known Parsonnet risk model for mortality from the late eighties, obesity, hypertension and diabetes mellitus were all risk factors for mortality.^1^ Opposed to that, none of these risk factors were considered important in the EuroSCORE, which was developed a decade later, from 19 000 patients in Europe who had had a CABG.^2^

In a recent study of 10 000 patients, obesity was not a risk factor for immediate mortality after CABG.^3^ Large studies are required to have the power to reach statistical significance and to demonstrate the influence of such co-morbidities. Combining 146 000 patients from various hospitals showed a mortality of 3.7% for patients with diabetes mellitus and 2.7% for those without diabetes mellitus.^4^

According to the Society of Thoracic Surgeons’ database, the odds ratio for mortality for patients with diabetes mellitus and hypertension is 1.3 and 1.2, respectively, compared to a redo CABG with an odds ratio of 3.1.^5^ A combination of these risk factors could strengthen their individual statistical power.

A triad of obesity (in particular central obesity), hypertension and diabetes mellitus fulfils the criteria for the metabolic syndrome. Dyslipidaemia is the fourth factor for diagnosing the metabolic syndrome.

In 1988 Gerald Reaven attributed the irregularities associated with the metabolic syndrome to insulin resistance.^6^ Abdominal fat functions as an endocrine organ that secretes pro-inflammatory adipokines, which could be the underlying pathophysiology for insulin resistance.^7^ C-reactive protein (CRP) is a marker of subclinical inflammation and a predictor of coronary incidents.^8^ Furthermore, central obesity leads to an increase in CRP. In fact, as the various components of the metabolic syndrome are added, the CRP increasingly rises.^9^

If a patient has an underlying inflammatory condition, as with the metabolic syndrome, and is subjected to a further inflammatory insult during cardiopulmonary bypass, it is postulated that the risk for mortality and morbidity could be higher. This was confirmed by a study in 2007. The prevalence of the metabolic syndrome among these 5 300 patients was 46%. The relative risk for mortality for patients with the metabolic syndrome was 3.04 (95% CI: 1.73–5.32; *p* = 0.0001). Obesity and diabetes mellitus, as single risk factors, could not be established as independent hazards for mortality.^10^ Morbidity was also more prominent among patients with the metabolic syndrome.

A year later another study came to a different conclusion. The metabolic syndrome had no impact on survival after treatment. These patients were treated with medication (*n* = 516), by percutaneous coronary intervention (*n* = 1 274), and CABG (*n* = 1 096).^11^

The prevalence of the metabolic syndrome is high. Among United States adults older than 20 years it is 24%, increasing with age to 40% among 60-year-olds.^12^ But it is not only a problem of the West. China experiences an epidemic of obesity, with the metabolic syndrome present in 13% of adults.^13^ In South Africa the prevalence is unknown, but from a study done in the Free State province among blacks, it could be as high as 31%, using the WHO criteria.^14^

A study was therefore undertaken to investigate the consequences of the metabolic syndrome on a local population of CABG patients. This was done to establish its effect on the pre-operative risk factors for mortality as well as on the outcome after CABG.

## Methods

The study was an analytical cohort study. The target population was all patients who had had a CABG done by one surgeon (MJS) at the Mediclinic Bloemfontein between November 2000 and October 2010. The metabolic syndrome was defined according to the criteria set by the International Diabetes Federation (IDF) in 2005.^15^ When the body mass index (BMI) was < 30 kg/m^2^ but hypertension, diabetes mellitus and dyslipidaemia were present, the definition from the National Cholesterol Education Programme Adult Treatment Panel (ATP III) in 2001 was used^16^ (Table 1). Central obesity was assumed with a BMI ≥ 30 kg/m^2^, which is acceptable according to IDF criteria.

**Table 1.** Criteria For The Metabolic Syndrome

*ATP III (2001)*	*IDF (2005)*
Three or more:	Central obesity
1. Abdominal obesity:	Waist circumference, ethnicity specific
waist circumference	plus any two:
≥ 94–102 cm (males)	1. Triglycerides:
≥ 80–88 cm (females)	≥ 1.7 mmol/l or specific treatment
2. Triglycerides:	2. HDL-C:
≥ 1.7 mmol/l	< 1.03 mmol/l (males)
3. HDL-C:	< 1.29 mmol/l (females)
< 1.03 mmol/l (males)	or specific treatment
< 1.29 mmol/l (females)	3. Hypertension:
4. Hypertension:	systolic ≥ 130 mmHg
systolic ≥ 130 mmHg	or diastolic ≥ 85 mmHg
or diastolic ≥ 85 mmHg	specific treatment
5. Fasting blood glucose:	4. Fasting plasma glucose:
≥ 6.1 mmol/l	≥ 5.6 mmol/l
	or previously diagnosed type 2
	diabetes

HDL-C: high-density lipoprotein cholesterol.

To ascertain the effect of the metabolic syndrome on the pre-operative risk factors for mortality after CABG, the seven core risk factors from a previous study were used and adapted.^17^ These risk factors were older age, female gender, re-operation, left ventricular ejection fraction ≤ 40%, critical left main stem disease, number of bypasses as an indication of disease severity, and urgency of operation. In this study, urgency was assumed when the operation was done from the coronary care unit with or without an intra-aortic balloon pump (IABP). These patients included those admitted with unstable angina and acute myocardial infarction.

Further information that was gathered included the older additive EuroSCORE for each patient and renal function according to the shortened Modification of Diet in Renal Disease formula (sMDRD).^18^ Patients with chronic kidney disease grade III were also documented.

Postoperative data that were evaluated were the Society of Thoracic Surgeons’ major negative outcomes: re-exploration, permanent stroke, renal impairment (new dialysis or 50% rise in serum creatinine level from pre-operative value), mechanical ventilation longer than 48 hours, and deep sternal infection, but for this study rewiring of the sternum for dehiscence was considered deep sternal infection.^19^ Patients who were discharged, but re-admitted within six weeks for sternal rewiring were considered part of the study. Other information obtained from patient records included the mediastinal drainage, homologous red blood cell transfusion, in-hospital mortality, and length of stay (LOS).

Patients were excluded from this study if it was not possible to make a diagnosis of the metabolic syndrome. Those patients who had a major procedure combined with the CABG, patients who were on pre-operative dialysis, and those who died on the operating table were also excluded. The study group was divided between patients with the metabolic syndrome and those without the metabolic syndrome. A group without central obesity, hypertension or diabetes mellitus was also identified.

## Statistical analysis

All the data were analysed by the Department of Biostatistics at the University of the Free State. Numerical data are expressed as means and ranges. Categorical variables are indicated in percentages. Differences were assessed using chi-square tests, Fisher exact tests, *t*-test or Kruskall-Wallis tests depending on the data type. This study was approved by the Ethics Committee of the Faculty of Health Sciences at the University of the Free State, Bloemfontein. All data were treated anonymously.

## Results

The initial study population was 1 475 patients. Unfortunately 495 patients had insufficient information to diagnose them with the metabolic syndrome or definitely exclude the metabolic syndrome. Ninety-three patients had another major cardiac procedure with the CABG and were not considered for the study. Three patients had an associated malignant resection at the time of cardiac surgery. Seven patients who had been on renal dialysis before the surgery were excluded, as were the four patients who died in theatre and could not be evaluated postoperatively.

From the remaining patients, 370 had the metabolic syndrome (322 according to the IDF criteria) and 503 patients did not meet the criteria. The prevalence of the metabolic syndrome among this study group was 42%. Of the group without the metabolic syndrome, 319 had no central obesity, hypertension or diabetes mellitus. On the other hand, 169 patients from the non-metabolic syndrome group had at least one of these three criteria and 15 had two criteria of the metabolic syndrome. However, three criteria are needed to diagnose the metabolic syndrome.

Table 2 summarises the results for the two groups, patients with the metabolic syndrome and those without the metabolic syndrome. The gender distribution was equal between the groups. Although the metabolic syndrome patients were slightly younger (median 59 years) than the non-metabolic syndrome group (median 61 years), this did not reach statistical significance.

**Table 2.** Results Of The Metabolic Syndrome And Non-Metabolic Syndrome Patients

	*Metabolic syndrome (n = 370)*		*Non-metabolic syndrome (n = 503)*		p-*value*
Age (mean)	59.2	59 median	60.4	61 median	0.0811
Gender, male:female	292:78	21.1% female	390:113	22.5% female	0.6250
Re-operation	42	11.4%	52	10.3%	0.6331
LVEF ≤ 40%	22	5.9%	22	4.4%	0.2940
Main stem	72	19.5%	90	17.9%	0.5562
Bypasses (mean number)	2.63	3 median	2.71	3 median	0.2318
Urgency	250	67.6%	381	75.7%	**0.0076**
EuroSCORE (mean)	3.26	3 median	3.61	3 median	**0.0494**
sMDRD ml/min (mean)	76.2		76.1		0.9960
CKD III	69	18.6%	90	17.9%	0.7749
Re-exploration	5	1.4%	15	3.0%	0.1115
Permanent stroke	3	0.8%	3	0.6%	0.7023
Renal impairment	42	11.4%	42	8.3%	0.1017
Ventilation > 48 hours	10	2.7%	8	1.6%	0.2531
Re-sternal wiring	6	1.6%	5	1.0%	0.5418
Total number of patients with any of the above 5	59	15.9%	62	12.3%	0.1271
Mortality	7	1.9%	8	1.6%	0.7348
Mortality + morbidity	58	16.5%	64	12.7%	0.0938
Mediastinal drainage (ml) (mean)	624		670		0.3420
RBC (units/patient) (mean)	0.4	0 median	0.73	0 median	**0.0012**
LOS days (mean)	5.9	5 median	5.8	5 median	**< 0.0001**

LVEF: left ventricular ejection fraction; sMDRD: shortened Modified Diet in Renal Disease; CKD III: chronic kidney disease grade III; LOS: length of stay; RBC: red blood cells.

As far as the other risk factors for mortality after CABG are concerned, there was no difference with regard to redo CABG, poor ventricular function, main stem lesion and number of bypasses. However, the metabolic syndrome group was operated on less urgently, as 67.6% were operated from the coronary care unit compared to 75.7% of the non-metabolic syndrome group (*p* = 0.0076).

The mean EuroSCORE also differed statistically (*p* = 0.0494). The metabolic syndrome group had a mean EuroSCORE of 3.26 (median 3) and the non-metabolic syndrome group 3.61 (median 3). The mean sMDRD was 76.2 and 76.1 ml/min for the two groups, respectively. The percentage of patients who had advanced to stage III chronic disease was almost the same (18.6 vs 17.9%).

Postoperatively, the mortality was similar for the two groups, with 1.9% of patients with the metabolic syndrome and 1.6% without the metabolic syndrome who died during their hospital stay. The re-exploration rate, percentages of patients with a permanent stroke, those with renal impairment, those with long mechanical ventilation periods, and patients who required rewiring of a dehisced sternum were similar in the two groups. Even if the total number of patients with morbidities were compared (15.9 vs 12.3%; *p* = 0.1271) the outcome was still matching.

The mean mediastinal drainage was almost the same in the two groups (624 and 670 ml, respectively). Homologous blood transfusion was less in the metabolic syndrome group, with a mean of 0.4 units per patient compared to the 0.73 units per patient from the non-metabolic syndrome group (*p* = 0.0012). Patients from the metabolic syndrome group stayed a mean of 5.9 days (median 5) and those from the non-metabolic syndrome group 5.8 days (median 5). This difference reached a *p*-value of < 0.0001 and therefore the metabolic syndrome patients had a more prolonged hospital stay.

## Discussion

The metabolic syndrome increases the risk of developing coronary heart disease. In the Atherosclerosis Risk in Communities study, 23% of the 12 000 patients had the metabolic syndrome without diabetes mellitus and existing cardiovascular disease. Over an average of 11 years, the men were 1.5 times and women twice as likely to develop coronary artery disease.^20^

The impact of the metabolic syndrome on the population was also addressed by Shaista Malik. In a cohort of 6 255 adult patients representing 16 million North Americans, 26% had the metabolic syndrome. The metabolic syndrome strongly predicted coronary heart disease, cardiovascular disease and all-cause mortality and more so than the individual components of the syndrome.^21^

For this study both the ATP III and IDF (2005) criteria were used. Since the completion of this series, the IDF has adapted its criteria. Central obesity is no longer an obligatory component, but it is one of five criteria, of which three constitute diagnosis of the metabolic syndrome.^22^ Central obesity is defined by waist circumference, but this is gender and ethnicity specific. Waist circumference was not available in our population but body weight and height were, from which the BMI could be derived. With a BMI of ≥ 30 kg/m^2^, 85% of men would have had a waist circumference of at least 102 cm and 98% of women a waist circumference of 88 cm and more.^23^

Patients without the metabolic syndrome might still have some of the components of the metabolic syndrome, of which each one is a risk factor for coronary artery disease. In the Framingham Heart study it was the triad of central obesity, hypertension and diabetes mellitus which had the highest risk for cardiovascular disease and mortality.^24^ Of those patients without the metabolic syndrome, 319 could be identified without a BMI ≥ 30 kg/m*2*, hypertension or diabetes mellitus. However, they could still have had dyslipidaemia. Even so, in this so-called ‘clean’ group, underlying undiagnosed diabetes mellitus could have been present.

In a study on the diagnostic value of haemoglobin A^1c^ and fasting plasma glucose levels in CABG patients with undiagnosed diabetes mellitus, 60% of patients who were initially admitted without diabetes mellitus were found to have diabetes one month after surgery.^25^ A pure non-metabolic syndrome group of patients is therefore hard to identify in sufficient numbers among patients with coronary artery disease.

In our study, the prevalence of 42% of patients with the metabolic syndrome correlates with others studies where the metabolic syndrome and CABG were investigated (46, 47, 51%).^10^, ^29^,^30^ However, in an overview of the metabolic syndrome in young South African Asian patients with myocardial infarction, the prevalence of the metabolic syndrome, depending on the definition, was as high as 69%.^26^

The perception was that patients with the metabolic syndrome might be younger. The median age difference was in fact two years, but this did not reach statistical significance. However the difference in the EuroSCORE was important. Age older than 60 years adds one percentage point to the EuroSCORE and this age difference below and above the age of 60 years probably contributed to the difference in EuroSCORE.

The metabolic syndrome made no impact on the pre-operative critical core risk factors for mortality after CABG; in fact it was patients without the metabolic syndrome who were operated on more urgently than patients with the metabolic syndrome. A possible explanation for this observation might be that patients with the metabolic syndrome have underlying risk factors for coronary artery disease such as hypertension, diabetes mellitus and dyslipidaemia and therefore are better followed up and intervention occurs sooner on a more elective basis. The lack of effect of the metabolic syndrome on renal function pre-operatively was surprising.

The postoperative outcome demonstrated hardly any difference between the two groups. The percentage of explorations for excessive mediastinal drainage was more than double in the non-metabolic syndrome group (3.0 vs 1.4%), but this did not reach statistical significance. In a study on the effect of obesity on the outcome after CABG, the rate of re-operation because of bleeding was double (*p* < 0.001) in the non-obese patients.^27^ The difference in actual volume of drainage was also not important.

A significant difference between the two groups was indeed observed in the number of units of homologous red blood cells transfused. Patients with the metabolic syndrome have higher BMI and therefore a higher blood volume and better reserves for loss of blood. It is also postulated that patients with the metabolic syndrome have less fibrinolytic activity and are more prone to hypercoagulability.^28^ This study could not demonstrate a difference between the two groups in the percentage of patients with stroke and renal impairment after CABG.

A Japanese study showed that on multivariate analysis, the metabolic syndrome had odds ratios of 2.47 (95% CI: 1.22–4.99; *p* = 0.012) for postoperative stroke and 3.81 (95% CI: 1.42–10.3; *p* = 0.008) for postoperative renal failure.^29^ The group of 319 patients without central obesity, hypertension or diabetes mellitus had less acute renal impairment after CABG (5.3 vs 11.4% with the metabolic syndrome; *p* = 0.0035). If the metabolic syndrome group was compared with this ‘clean’ group, the occurrence of mortality plus major morbidity was significantly different (15.7 vs 9.1 %; *p* = 0.0095), but it is probably the renal outcome that drove this finding. As far as the metabolic syndrome and non-metabolic syndrome per definition is concerned, this study could not show an effect of the metabolic syndrome on mortality and major morbidity.

The length of hospital stay was statistically longer in patients with the metabolic syndrome although the median stay was similar. This is explained by the fact that the group without the metabolic syndrome had more patients staying four days or less, than the group with the metabolic syndrome (41 vs 27%). Although statistically important, the clinical effect of 0.1 day (difference in mean) longer or shorter stay is negligible.

In another smaller study (657 patients) than this one, the hospital stay was also longer for patients with the metabolic syndrome (8.3 vs 6.6 days; *p* = 0.003). In addition, this smaller study also found no difference for morbidity, although female patients with the metabolic syndrome had a higher mortality rate.^30^

## Conclusions

This study confirmed the high prevalence of the metabolic syndrome among patients who undergo CABG. It failed to demonstrate an effect of the metabolic syndrome on the pre-operative risk factors for CABG mortality. In fact, patients with the metabolic syndrome were operated on more electively than those without the metabolic syndrome. The only negative outcome in patients with the metabolic syndrome was their longer stay, although not clinically important. The ‘benefit’ of having the metabolic syndrome was the lower risk for homologous blood transfusion after surgery.

This study can contribute numbers for future analysis of the influence of the metabolic syndrome in patients undergoing CABG. Until then, Simons *et al*. might be correct that the metabolic syndrome as they see it should only been regarded as an educational tool and has limited practical value for diagnosis or management.^31^
